# Floral and Vegetative Micromorphology of Selected Species of *Anoectochilus* (Orchidaceae): Insights into Trichome Diversity and Peloria

**DOI:** 10.3390/biology15161341

**Published:** 2026-08-08

**Authors:** Adrianna Maria Kosiróg-Ceynowa, Magdalena Narajczyk, Dorota Łuszczek, Sławomir Nowak

**Affiliations:** 1 Department of Plant Taxonomy and Nature Conservation, University of Gdańsk, Wita Stwosza 59, 80-308 Gdansk, Poland; adrianna.kosirog@phdstud.ug.edu.pl; 2 Bioimaging Laboratory, Faculty of Biology, University of Gdańsk, Wita Stwosza 59, 80-308 Gdansk, Poland; magdalena.narajczyk@ug.edu.pl (M.N.); dorota.luszczek@ug.edu.pl (D.Ł.)

**Keywords:** Goodyerinae, floral micromorphology, scanning electron microscopy, trichomes, stomata, papillae, peloria, taxonomy

## Abstract

The genus *Anoectochilus* (Orchidaceae) is characterized by considerable floral diversity, yet its micromorphology remains poorly understood. This study aimed to investigate the distribution and morphology of micromorphological structures in selected representatives of the genus, with particular emphasis on the putatively peloric species *Anoectochilus papuanus*. The study was designed as a preliminary qualitative survey, with each species represented by a single specimen. Flowers of *A. papuanus*, *A. formosanus*, and *A. regalis*, as well as leaves of *A. papuanus* and *A. formosanus* and the inflorescence rachis of *A. papuanus*, were examined using scanning electron microscopy. Trichomes, stomata, and papillae were documented on floral organs, ovaries, leaves, and the inflorescence rachis. Three principal trichome types were distinguished: oblate, pyriform, and conical. *Anoectochilus papuanus* exhibited the greatest observed trichome diversity, whereas only oblate trichomes were observed in the available material of *A. regalis*, although preservation-related deterioration prevented its comprehensive examination. Stomata were observed on floral organs and leaves, with actinocytic, desmocytic, and pericytic types recorded. Papillae occurred on different floral structures and varied in their distribution among the examined species. The labellum of *A. papuanus* differed from the labella of the remaining species by the presence of stomata and a wrinkled epidermis, resembling the petal surfaces. These observations are consistent with previous interpretations of *A. papuanus* as a peloric form and document previously unreported micromorphological variation in *Anoectochilus*. However, the taxonomic and functional significance of the observed characters requires confirmation using broader sampling and quantitative analyses.

## Simple Summary

Orchids are one of the most diverse groups of flowering plants, but many details of their surface structures remain poorly studied. These microstructures can help scientists understand how species differ from one another and may provide useful information for plant identification. In this study, we examined flowers of three species and vegetative organs available from two species of jewel orchids belonging to the genus *Anoectochilus* using scanning electron microscopy. The study was preliminary and qualitative, and each species was represented by one plant specimen. We documented the occurrence of hair-like structures, stomata, and epidermal projections on different plant organs. *Anoectochilus papuanus* showed the greatest observed diversity of structures and differed from the other examined species in several features of its labellum. These observations are consistent with previous suggestions that this species is a peloric form, in which the labellum resembles the petals rather than showing the structure typical of the genus. The results also indicate that some features, especially the shape of trichomes and the arrangement of stomata on leaves, may be useful in future comparisons among species. This study provides the first overview of micromorphological diversity in *Anoectochilus* and creates a foundation for future taxonomic and evolutionary research on this group of orchids. However, broader studies involving more species and individuals are required to confirm the significance of the observed differences.

## 1. Introduction

Orchidaceae is one of the largest and most diverse families of flowering plants. Considerable diversity among orchids can also be observed in the micromorphology of floral and vegetative structures. Micromorphological features in orchids have been studied mainly in relation to pollination biology and their potential taxonomic value, including their application in intrageneric classification. Studies on floral micromorphology have been conducted in representatives of the genera *Scuticaria* Lindl. and *Dichaea* Lindl. [1], as well as in *Bulbophyllum* Thouars sect. *Didactyle* (Lindl.) Cogn. [2], where significant differences in epidermal structures were documented despite their presumed conservative character. Davies et al. [3] and Mytnik et al. [4] investigated micromorphological structures occurring on perianth segments in selected representatives of the subtribe Polystachyinae Schltr. Furthermore, Davies and Stpiczyńska [1,5,6] examined the potential role of floral micromorphological structures in pollination processes in species of *Scuticaria*, *Dichaea*, *Mormolyca* Fenzl., *Maxillaria* Ruiz & Pav., and representatives of the subtribe Bifrenariinae Dressler. Similar studies were carried out by Kowalkowska et al. [7,8,9,10] in representatives of Bulbophyllinae Schltr., Pleurothallidinae Lindl., and species of *Epipactis* Zinn. Additional investigations were conducted by Lipińska and Kowalkowska [11] in *Maxillaria* and by Lipińska et al. [12] in three species of *Maxillariella* M.A. Blanco & Carnevali. Besi et al. [13,14] analyzed the occurrence of papillae, trichomes, and epicuticular waxes on floral segments of *Vanda* R.Br. and *Paphiopedilum* Pfitzer.

Other studies have focused on the micromorphology of the floral spur, which is considered an important structure influencing pollinator behavior in orchids [15]. Spur morphology has been investigated in representatives of *Anacamptis* (L.) Rich., *Orchis* L., *Gymnadenia* R.Br., *Dactylorhiza* Neck. ex Nevski, *Platanthera* Rich., *Pseudorchis* Ség., *Neolindleya* Kraenzl., and *Galearis* Raf.

The orchid genus *Anoectochilus* Blume is one of the largest genera within the subtribe Goodyerinae Klotzsch. Its representatives are terrestrial plants characterized by sympodial growth and creeping rhizomes. Species of the genus occur in tropical regions of Southeast Asia and Australasia, ranging from Sri Lanka and India to Japan, Indonesia, Papua New Guinea, New Caledonia, Timor, the Solomon Islands, Fiji, Vanuatu, and Samoa. In Australia, representatives of the genus are found mainly in northeastern Queensland [16]. Most species inhabit the forest floor, growing in substrates rich in leaf litter and humus. Leaves are spirally arranged and vary in colour from dark green to brown or nearly black, usually with contrasting venation. The inflorescence is simple and typically bears a few flowers [16]. Floral segments are distinctly differentiated. Sepals and petals are generally simple, entire, and occasionally concave, whereas the lip is highly modified, often lobed and ornamented with numerous linear fringes. An exception is *A. papuanus* (Schltr.) W. Kittr., in which the lip is simple and petal-like which had led to its previous interpretation as a peloric form within the genus [17]. Various trichomes have been reported on floral parts, as well as on the ovary and inflorescence axis. However, despite the considerable morphological diversity observed within the genus, no comprehensive study on the micromorphology of *Anoectochilus* species has yet been conducted. Moreover, within Goodyerinae, micromorphological investigations remain relatively scarce. Previous studies in the subtribe have focused on floral structures in *Aspidogyne* Garay, *Microchilus* C. Presl, and *Zeuxine* Lindl. [18], as well as on seed micromorphology in *Platylepis* A. Rich. [19] and *Cheirostylis* Blume [20].

**Figure 1:**
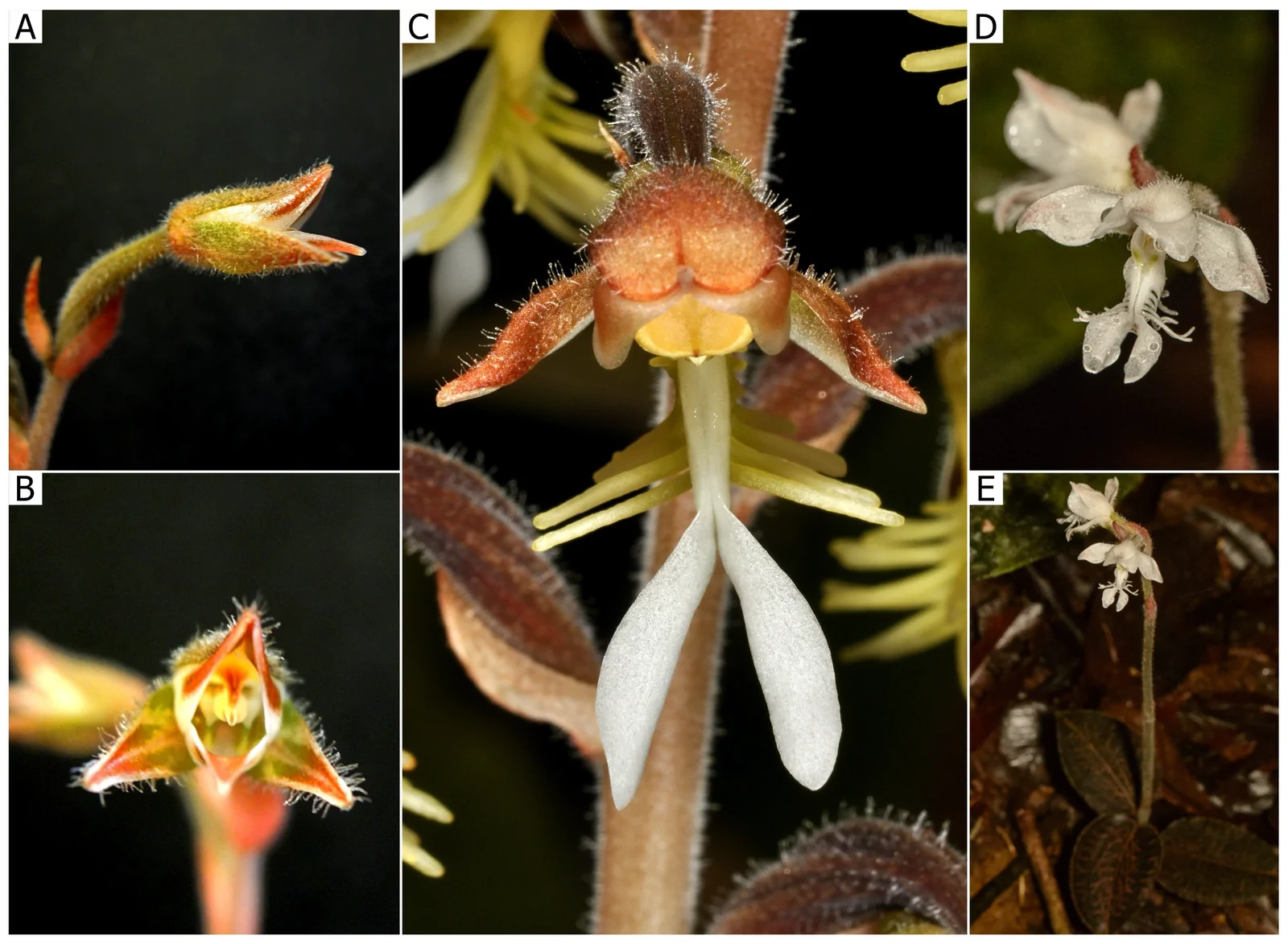
Examined species of *Anoectochilus*: *A. papuanus* (Schltr.) W. Kittr. ((**A**,**B**); photographed by Sławomir Nowak), *A. formosanus* Hayata ((**C**); photographed by Ron Parsons), and *A. regalis* Blume ((**D**,**E**); photographed by Arthur Yin).

Therefore, the present study aims to fill the existing gap in knowledge concerning the floral micromorphology of the genus *Anoectochilus*. Particular attention is given to *Anoectochilus papuanus*, regarded as a probable peloric form within the genus, in order to evaluate the extent of its micromorphological distinctiveness in comparison with other representatives of the genus (Figure 1).

## 2. Materials and Methods

The research material consisted of three species of the genus *Anoectochilus* Blume. *Anoectochilus papuanus* (Schltr.) W. Kittr. and *A. formosanus* Hayata were represented by living specimens, whereas material of *A. regalis* Blume was obtained from the spirit collection and had been preserved in Kew Mixture (53% ethyl alcohol, 37% water, 5% formaldehyde, and 5% glycerol). The material was made available from the living plant collection and the spirit collection in the herbarium of the Faculty of Biology, University of Gdańsk. For each species, one flower from a single specimen was examined. In addition, a leaf fragment was collected from the same specimen of each of the two living species, *A. papuanus* and *A. formosanus*. A fragment of the inflorescence rachis was examined only in *A. papuanus*. Thus, all observations for each species were based on material obtained from a single individual (Tables S1–S3).

Fresh material was fixed for 24 h at room temperature in 2.5% (*w*/*v*) paraformaldehyde (PFA) and 2.5% (*v*/*v*) glutaraldehyde (GA) in 0.1M cacodylate buffer. The material was subsequently washed twice in 0.1 M cacodylate buffer for 15 min at room temperature and dehydrated through a graded ethanol series. Samples were dried with liquid CO_2_ using an Emitech Critical Point Dryer K850 (Emitech Ltd., Ashford, Kent, UK). The dried material was mounted on SEM stubs and sputter-coated with an approximately a 10 nm layer of gold using an SPI-Module Sputter Coater (Structure Probe, Inc, West Chester, PA, USA). All preparations were observed and photographed using a Philips XL-30 scanning electron microscope (Philips Electron Optics, Eindhoven, The Netherlands) operated at an accelerating voltage of 15 or 20 kV and equipped with a secondary electron detector.

The study was designed as a qualitative and exploratory survey. Micromorphological structures were characterized descriptively, without quantitative comparisons or statistical analyses, because each species was represented by a single individual.

Trichome types were distinguished qualitatively based on the morphology of the terminal cell: flattened or rounded terminal cells forming a cap were classified as oblate, pear-shaped terminal cells as pyriform, and elongated, tapering terminal cells as conical.

## 3. Results

Micromorphological structures were examined in three species of the genus *Anoectochilus* Blume: *A. papuanus* (Schltr.) W. Kittr., *A. formosanus* Hayata, and *A. regalis* Blume. Floral segments and ovaries were analyzed in all three examined specimens. Additionally, leaf surfaces of *A. papuanus* and *A. formosanus*, as well as the inflorescence rachis surface of *A. papuanus*, were investigated. Trichomes, stomata, and papillae were observed on the examined floral and vegetative organs. The material of *A. regalis* was affected by preservation-related deterioration, including the collapse of a large proportion of cells. As a result, detailed characterization of some structures, particularly their cellular organization and surface morphology, was not possible, although structures that remained recognizable could still be observed and documented. Consequently, the apparent absence of particular micromorphological structures in the examined material of *A. regalis* should not be interpreted as their confirmed absence from the species.

### 3.1. Floral Trichomes

Trichomes were present on the outer surface of the sepals and ovary in the examined material of *A. papuanus*, *A. formosanus*, and *A. regalis*. All observed trichomes were multicellular, elongated, and cylindrical, differing mainly in the morphology of the terminal cell. Three principal trichome types were distinguished: oblate, pyriform, and conical.

**Figure 2:**
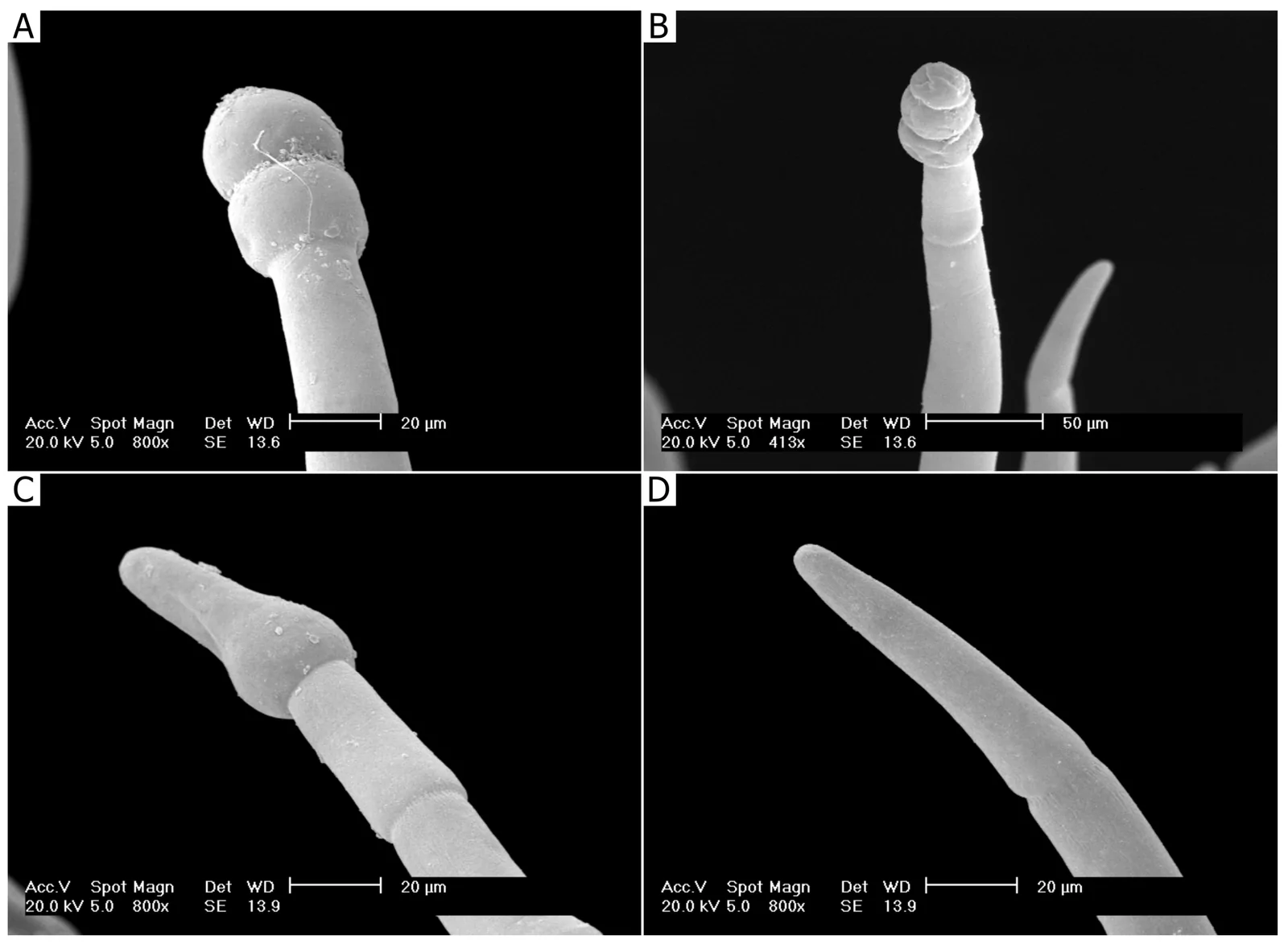
Representative floral trichome types observed in *Anoectochilus papuanus*: oblate trichomes with a terminal cap formed by two rounded, flattened cells (**A**) or three rounded, flattened cells (**B**), pyriform trichome (**C**), and conical trichome (**D**).

Oblate trichomes consisted of 4–6 cells and possessed a terminal cap formed by one to three rounded or flattened cells (Figure 2A,B); in some cases, two terminal cells were superimposed. Pyriform trichomes were composed of 3–4 cells and terminated in a pear-shaped apical cell (Figure 2C). Conical trichomes were composed of 4–5 cells and terminated in an elongated cone-shaped apical cell with a rounded apex (Figure 2D).

All three trichome types were observed in *A. papuanus* (Figure 2). In *A. formosanus*, conical and oblate trichomes were recorded. In *A. regalis*, only oblate trichomes were observed in the available material. However, preservation-related cell collapse prevented precise determination of the number of cells forming the terminal cap and may have limited the detection of other trichome types.

### 3.2. Stomata

**Figure 3:**
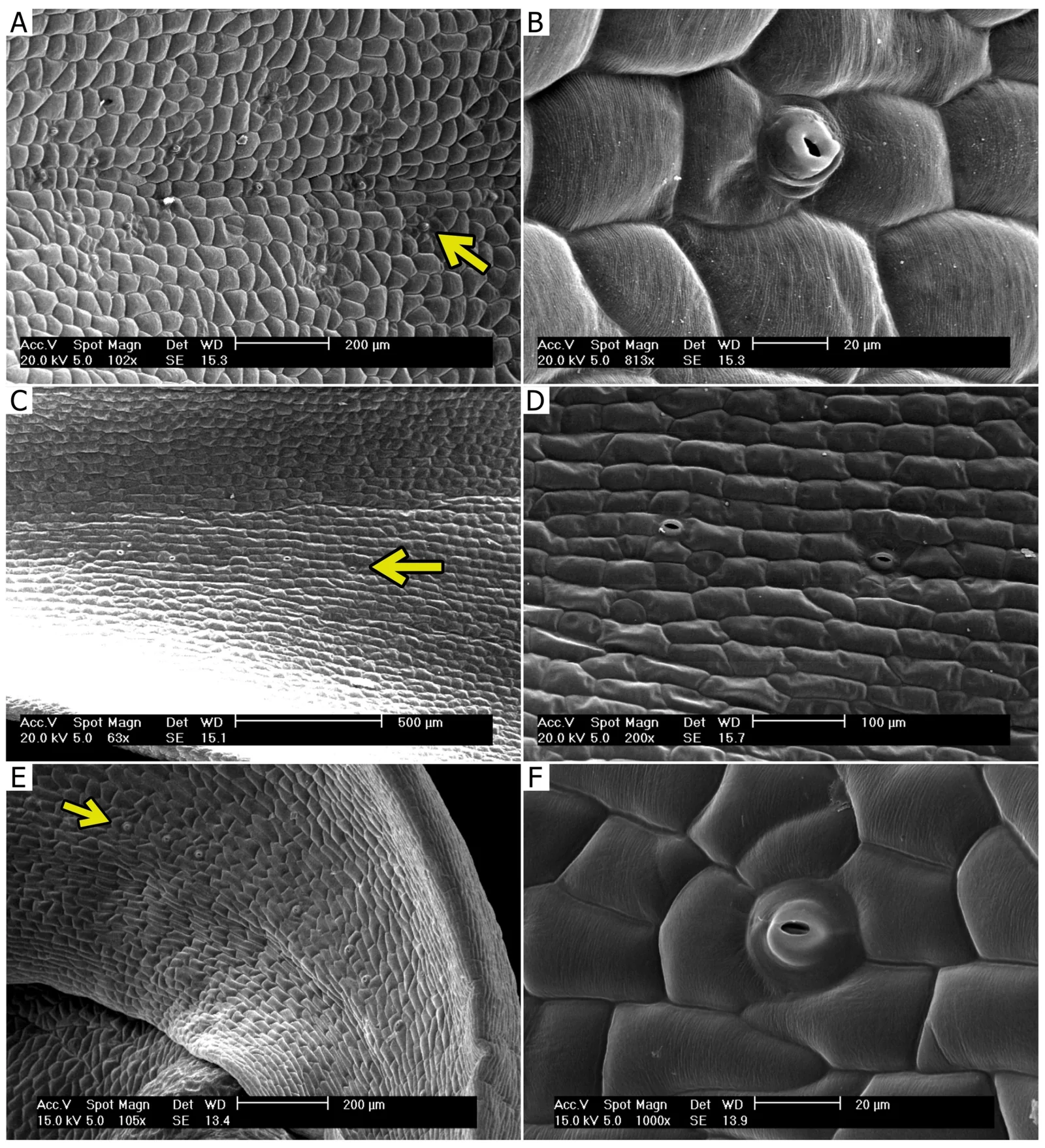
Distribution of stomata in *Anoectochilus papuanus* on the dorsal sepal (**A**) with an actinocytic stoma (**B**) and on the labellum (**C**) with a desmocytic stomata (**D**), and in *Anoectochilus formosanus* on the lateral sepal (**E**) with a pericytic stoma (**F**). Arrows indicate the locations of stomata.

Stomata were observed on the inner surface of the tepals in *A. papuanus* and *A. formosanus*, as well as on the inner surface of the labellum in *A. papuanus* (Figure 3). On the inner surface of the dorsal sepal of *A. papuanus*, stomata occurred along the main vein and were very sparsely distributed in the concave central region (Figure 3A). On the labellum, a single row of stomata was located in its slightly thickened central region (Figure 3C). Similarly, on the inner surface of the lateral sepal of *A. formosanus*, stomata occurred in a single row located in the central part of the organ (Figure 3E). Different stomatal types were recorded among the studied species and organs. In *A. papuanus*, actinocytic stomata were present on the dorsal sepal (Figure 3B), whereas desmocytic stomata occurred on the labellum (Figure 3D). In *A. formosanus*, pericytic stomata were observed on the lateral sepal (Figure 3F).

### 3.3. Papillae

**Figure 4:**
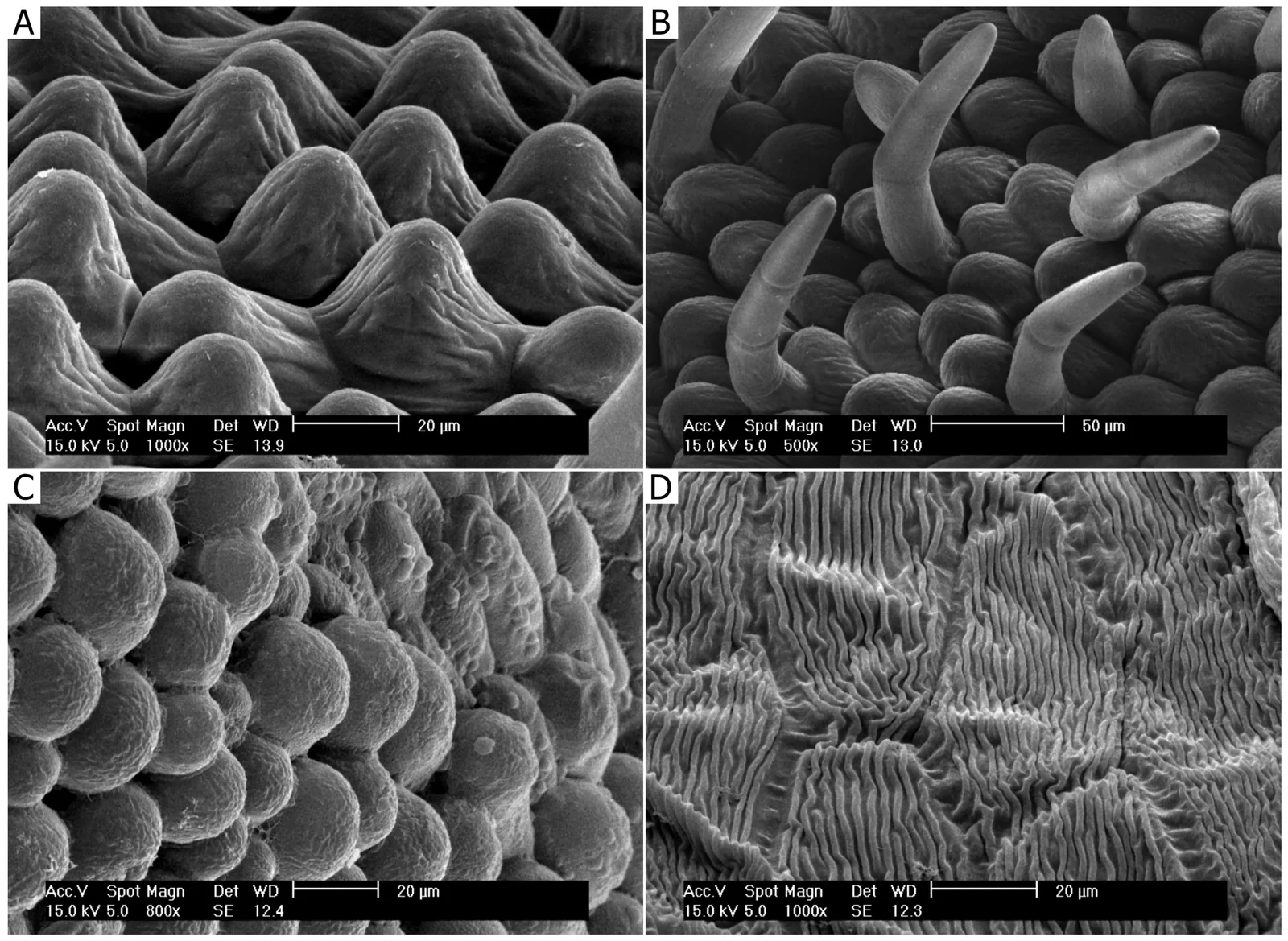
Micromorphological diversity of papillae in *Anoectochilus formosanus*: papillae present on the ovary (**A**), dorsal sepal, together with associated trichomes (**B**), and column (**C**), as well as column epidermal cells exhibiting undulate ridges (**D**).

Papillae were observed on the column of *A. papuanus*; on the ovary (Figure 4A), dorsal sepal (Figure 4B), column (Figure 4C), and labellum of *A. formosanus*; and on the ovary and labellum of *A. regalis*. In all studied species, the observed papillae were hemispherical. On the column surface of *A. formosanus*, epidermal cells with elongated, undulating hexagonal outlines were additionally observed (Figure 4D).

### 3.4. Leaf Micromorphology

**Figure 5:**
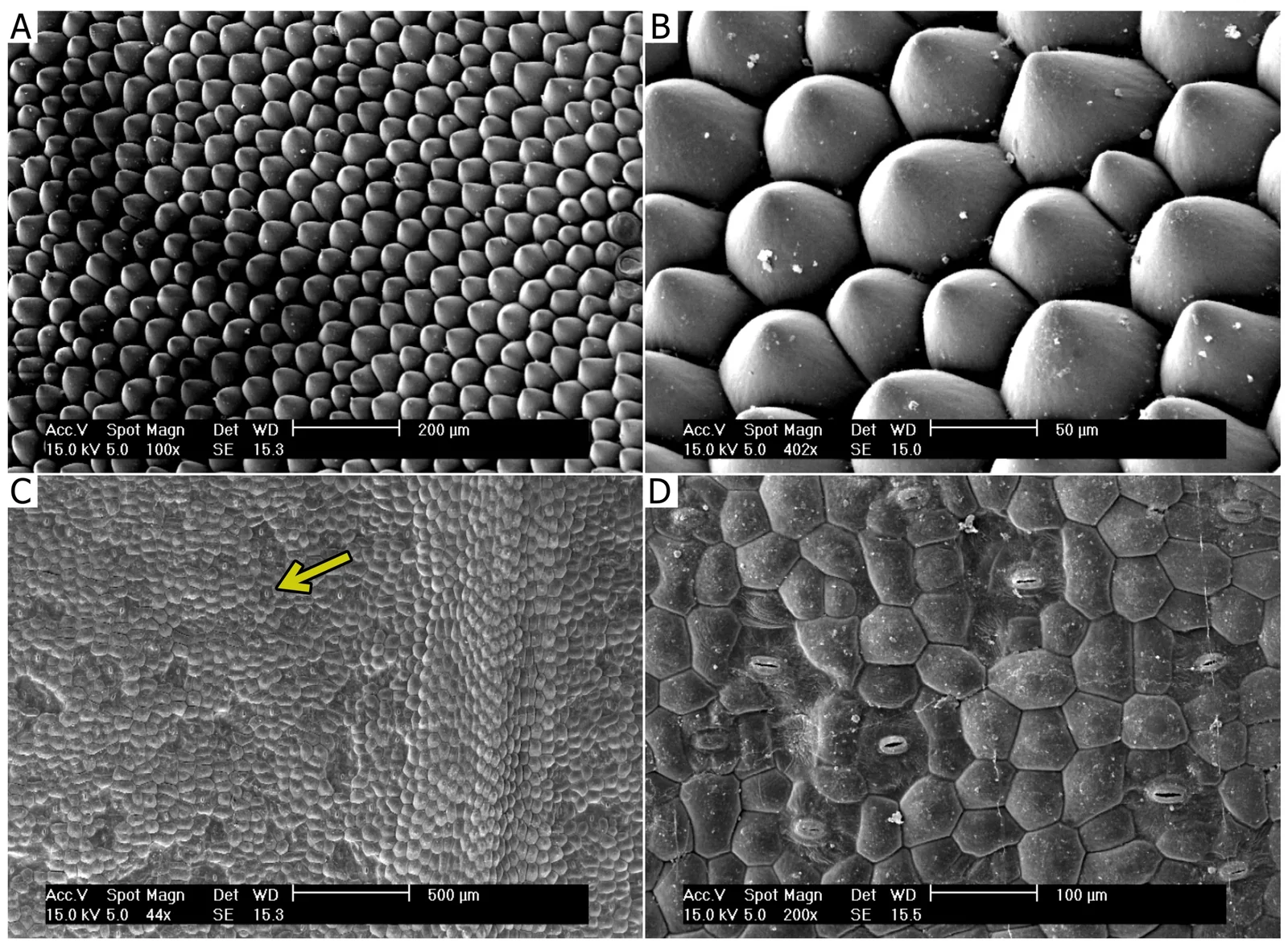
Micromorphology of the leaf epidermis in *Anoectochilus papuanus*: adaxial leaf surface (**A**) and a detail of the epidermal cells (**B**); abaxial leaf surface with a stomata (indicated by an arrow) (**C**) and a detail of the epidermal cells (**D**).

**Figure 6:**
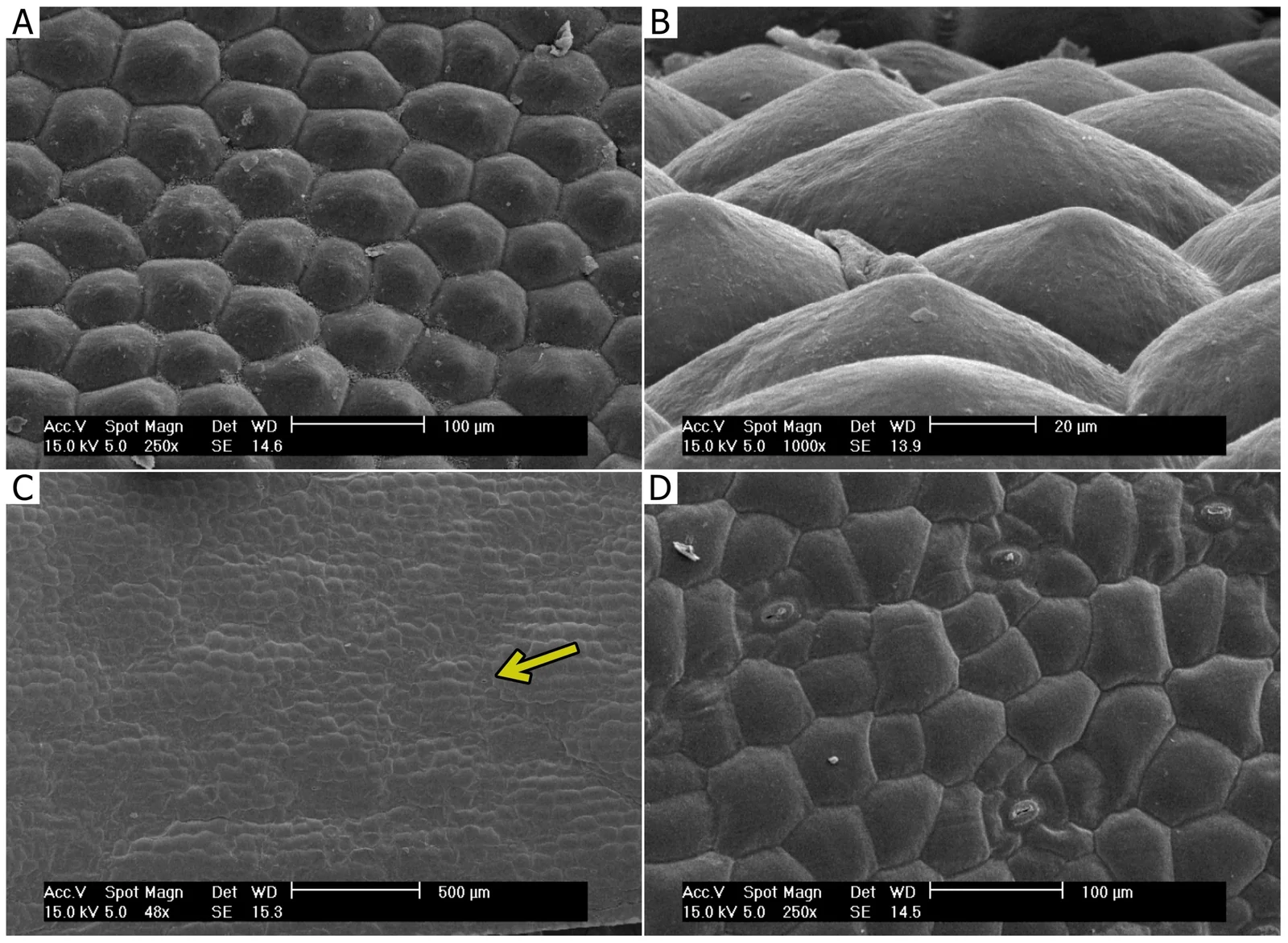
Micromorphology of the leaf epidermis in *Anoectochilus formosanus*: adaxial leaf surface (**A**) and a detail of the epidermal cells (**B**); abaxial leaf surface with a stomata (indicated by an arrow) (**C**) and a detail of the epidermal cells (**D**).

The adaxial and abaxial leaf surfaces of *A. papuanus* and *A. formosanus* showed differences in epidermal structure. On the abaxial surface, stomata were present in both species. In *A. papuanus*, stomata were actinocytic, whereas in *A. formosanus* they were pericytic. In both species, epidermal cells on the adaxial surface were conical in shape and showed a similar morphology (Figure 5 and Figure 6). However, the cells of *A. papuanus* appeared more tightly packed and had a more convex surface than those of *A. formosanus*. In contrast, the abaxial epidermis of both species was characterized by a wrinkled surface and irregularly arranged cells forming a mosaic-like pattern (Figure 5 and Figure 6).

### 3.5. Inflorescence Rachis

**Figure 7:**
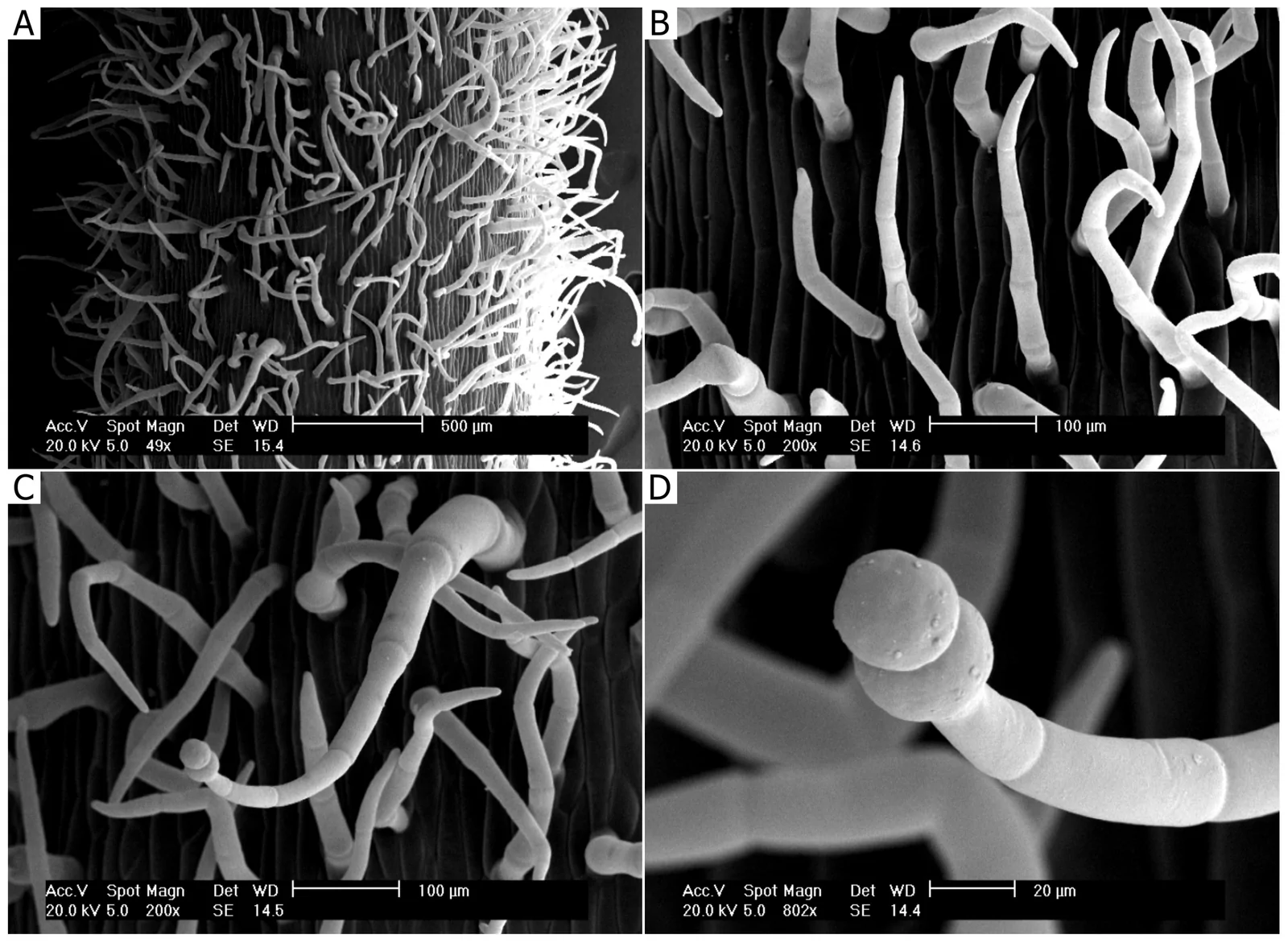
Micromorphology of the inflorescence rachis in *Anoectochilus papuanus*, showing the observed trichome types (**A**), with details of conical trichomes, including numerous hook-shaped forms (**B**), and an oblate trichome (**C**) with a close-up of its apical portion (**D**).

The inflorescence rachis was examined only in *A. papuanus*. The epidermis was smooth, and the cells were regularly arranged. Numerous multicellular trichomes were observed and classified into two morphological types: oblate and conical (Figure 7). Oblate trichomes were composed of 5–9 cells, and conical trichomes of 4–6 cells. Rachis trichomes were composed of a greater number of cells than the trichomes observed on floral organs, and many of them were hook-shaped.

## 4. Discussion

Among the studied representatives of the genus *Anoectochilus* Blume, both similarities and differences in the distribution and morphology of micromorphological structures were observed. *Anoectochilus papuanus* showed the most diverse combination of the observed micromorphological features and is regarded as a probable peloric form within the genus. In this species, the labellum resembles the petals and lacks a spur, in contrast to the remaining representatives of *Anoectochilus*. The presence of stomata and a distinctly wrinkled epidermal surface on the labellum of *A. papuanus* increases its micromorphological similarity to the petals and distinguishes the examined specimen from *A. formosanus* and *A. regalis*, in which the labellum surface was covered mainly by hemispherical papillae and lacked stomata. These features may indicate a partial morphological convergence between the labellum and petals in *A. papuanus* and are consistent with its previous interpretation as a peloric form. However, the present observations provide only an additional line of micromorphological evidence and are not sufficient to confirm the stability of peloria across individuals or populations. Nevertheless, the remaining floral segments of *A. papuanus* showed micromorphological similarity to those of the other examined species. Trichomes were the most frequently observed micromorphological structures and occurred on floral organs as well as on the ovary. All observed trichomes were multicellular and cylindrical, differing mainly in the morphology of the terminal cell.

Trichomes occurring on the outer floral surfaces of Goodyerinae are common and have been used as supplementary characters in species descriptions and identification. Similar structures have also been documented in other orchid groups, where they have been proposed to serve a variety of functional roles [4,21]. However, the functions of the trichomes documented here were not investigated in the present study. Among the studied taxa, oblate trichomes were the most common type, whereas conical and pyriform trichomes were observed only in some of the examined specimens. *Anoectochilus papuanus* exhibited the greatest diversity of trichome morphology, with all three recognized types observed in its flower, while only oblate trichomes were observed in the available material of *A. regalis*. However, the poor preservation of *A. regalis* may have prevented the recognition of other trichome types, and their absence should therefore not be regarded as a confirmed species-level character. The number of cells forming the trichomes remained relatively similar among the examined species, suggesting that the morphology of the apical cell may have greater potential taxonomic relevance than trichome size or cell number. This possibility requires confirmation using multiple individuals and broader taxonomic sampling.

The occurrence of stomata on floral organs was generally similar among the studied species, particularly in their concentration along the central region of the examined sepals. Differences in stomatal type were observed; however, their taxonomic significance appears to be limited because the same species may possess different stomatal arrangements on different floral organs. In contrast, stomatal arrangement on leaf surfaces may provide useful comparative information within the genus. *Anoectochilus papuanus* possessed actinocytic stomata, whereas *A. formosanus* showed pericytic stomata. Differences were also observed between the adaxial and abaxial leaf surfaces. The abaxial epidermis in both studied species was characterized by a wrinkled surface, mosaic-like cell arrangement, and the presence of stomata, whereas the adaxial epidermis consisted of regularly distributed conical cells. Such differentiation between the upper and lower leaf surfaces may reflect functional specialization associated with gas exchange and protection against excessive water loss. However, this possible functional interpretation was not tested in the present study. Moreover, leaves were available from only one individual of each of these two species and were not examined in *A. regalis*, the taxonomic consistency of these differences cannot yet be assessed.

Papillae were observed on different floral organs and varied in their distribution among the examined species. Although all observed papillae were hemispherical, their occurrence on the column, labellum, or ovary differed between taxa, suggesting that their distribution could provide potentially useful comparative characters. Similar micromorphological floral structures, including papillae and trichomes, have previously been reported in other orchid groups and are often discussed in the context of pollinator attraction and tactile guidance mechanisms [4,21,22]. The present study did not examine pollinator interactions, and these possible functions therefore remain hypotheses derived from previous studies. The diversity of multicellular trichomes observed in *Anoectochilus*, particularly the variation in terminal-cell morphology, has not been comprehensively documented within Goodyerinae.

The present study should be considered a preliminary qualitative contribution to the knowledge of micromorphological diversity in *Anoectochilus*, mainly because of the limited availability and condition of the examined material. Although floral structures and ovaries were studied in all three species, the available material was not fully equivalent among taxa. Leaf micromorphology could be examined only in *A. papuanus* and *A. formosanus*, whereas the inflorescence rachis was available only for *A. papuanus*. Consequently, no conclusions can yet be drawn regarding the leaf or rachis micromorphology of *A. regalis*. In this species, preservation-related cellular collapse caused considerable difficulties in interpreting some structures and prevented their full comparison with those of the remaining species. Although the specimen had been preserved in Kew Mixture, its condition was insufficient to maintain all cellular structures in an undisturbed state. Further studies based on fresh or adequately preserved material are therefore needed to re-examine this species and compare new observations with the present results. Nevertheless, the available observations of *A. regalis* remain valuable because micromorphological data for this species and for the genus as a whole are scarce.

Quantitative analyses and statistical comparisons were not performed because the study was designed as a qualitative, exploratory survey and each species was represented by a single individual. Although multiple structures could potentially be measured within each specimen, such measurements would not constitute independent biological replicates and would not support statistical inference at the species level. The present descriptions should therefore be treated as initial observations and hypotheses for further testing rather than as evidence of consistent interspecific differences.

Despite these limitations, the study provides the first comparative information on selected floral and vegetative micromorphological features in *Anoectochilus*. In Orchidaceae, floral surface structures such as trichomes, papillae, epidermal ornamentation, and stomata have been examined in relation to taxonomy and floral biology [4,13,14,21,22,23,24]. These structures may influence the surface texture of floral organs, particularly the labellum, and in some orchid groups have been associated with pollinator attraction, floral rewards, or tactile guidance of visiting insects. The stomata observed on floral organs are also noteworthy; however, their occurrence should not automatically be interpreted as evidence of the same physiological functions performed by stomata on leaves. In *Anoectochilus*, the functional significance of the documented structures remains unknown and requires targeted anatomical, developmental, and ecological investigation. Therefore, the micromorphological variation described here provides a starting point for future research on the biology, development, and taxonomy of the genus rather than definitive evidence of diagnostic or adaptive differentiation.

Overall, the results show variation in the distribution and morphology of selected micromorphological structures among the examined specimens of *Anoectochilus*. Some of these characters, particularly trichome morphology and leaf stomatal arrangement, may provide potentially useful comparative information. However, their taxonomic relevance remains preliminary. Broader studies incorporating more species, multiple individuals per species, equivalent sets of organs, and quantitative data are necessary to evaluate the consistency, systematic value, and possible application of these characters in the intrageneric classification of *Anoectochilus*.

## 5. Conclusions

This study provides the first qualitative and exploratory analysis of floral and vegetative micromorphology in selected species of *Anoectochilus*. Trichomes, stomata, and papillae were documented on floral organs, ovaries, leaves, and the inflorescence rachis, revealing both shared and variable patterns among the examined specimens. Among the examined taxa, *Anoectochilus papuanus* displayed the greatest diversity of observed trichome types and was distinguished by the presence of stomata and a wrinkled epidermis on the labellum. Because each species was represented by a single individual, these differences should not be interpreted as consistent species-level characteristics. These features increase the similarity between the labellum and petals and are consistent with previous interpretations of *A. papuanus* as a peloric form. However, the present observations provide only additional micromorphological support for this hypothesis and do not confirm the stability of peloria across individuals or populations.

The results indicate that certain micromorphological characters, particularly trichome morphology and leaf stomatal arrangement, may provide useful preliminary comparative information within the genus. However, the limited number of species and individuals examined, the unequal availability of organs, and the preservation-related deterioration of *A. regalis* prevent broader systematic conclusions. Future studies incorporating multiple individuals per species, broader and equivalent taxonomic sampling, quantitative anatomical analyses, and a phylogenetic framework will be necessary to evaluate the intraspecific variability, consistency, evolutionary significance, and potential taxonomic value of these characters across *Anoectochilus* and related genera of Goodyerinae.

## Data Availability

The original contributions presented in this study are included in the article/Supplementary Material. Further inquiries can be directed to the corresponding authors.

## Supplementary Materials

The following supporting information can be downloaded at: https://www.mdpi.com/article/10.3390/biology15161341/s1, Table S1: Micromorphology of *Anoectochilus papuanus* (Schltr.) W.Kittr.; Table S2: Micromorphology of *Anoectochilus formosanus* Hayata; Table S3: Micromorphology of *Anoectochilus regalis* Blume.

## Abbreviations

The following abbreviations are used in this manuscript:

SEM	scanning electron microscope
PFA	paraformaldehyde
GA	glutaraldehyde
