# Supplementary material for: Floral and Vegetative Micromorphology of Selected Species of Anoectochilus (Orchidaceae): Insights into Trichome Diversity and Peloria
Source: Biology (Basel). 2026 Aug 8;15(16):1341. doi: 10.3390/biology15161341 (PMC13509326; doi:10.3390/biology15161341)
Supplement: Supplementary file 1 [file biology-15-01341-s001.zip › Table S1 A.papuanus .pdf]

Table S1. Micromorphology of *Anoectochilus papuanus* (Schltr.) W.Kittr.

Floral structures

Structure	SEM Image 1	SEM Image 2	SEM Image 3
Lateral sepal	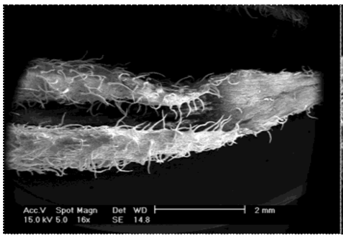	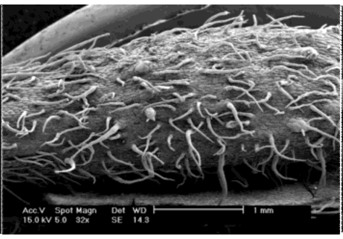	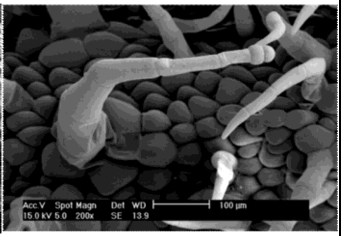
Gynostemium (column)	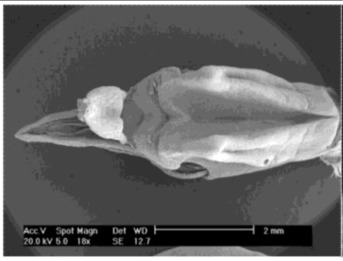	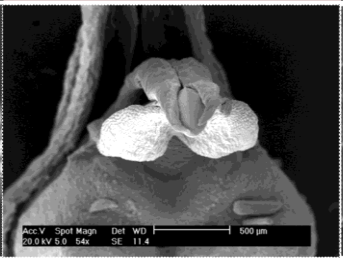	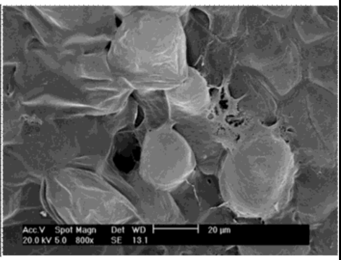
Dorsal sepal	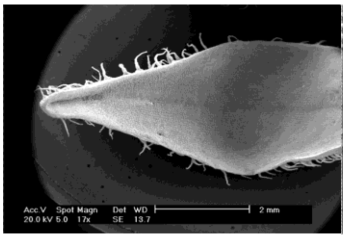	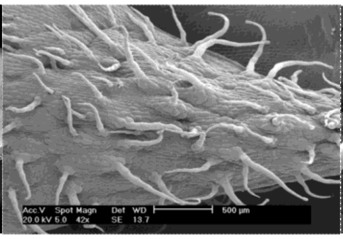	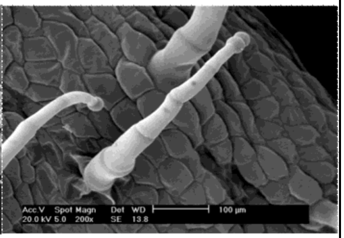
Labellum	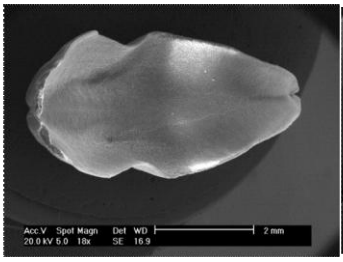	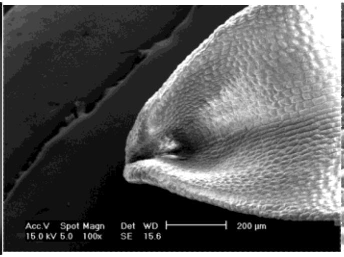	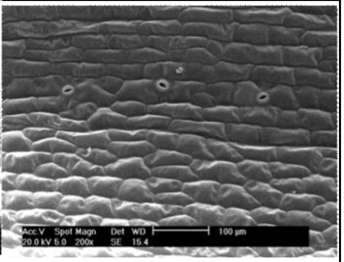
Ovary	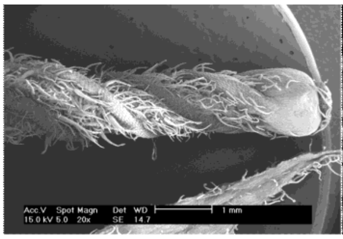	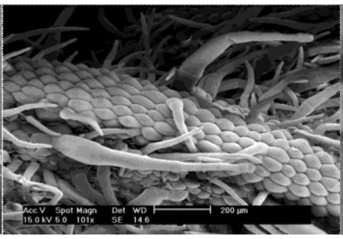	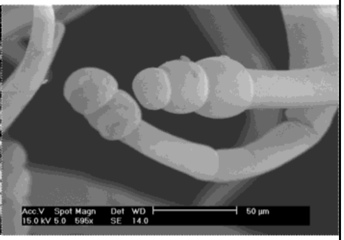

Vegetative structures

Structure	SEM Image 1	SEM Image 2	SEM Image 3
Abaxial leaf epidermis	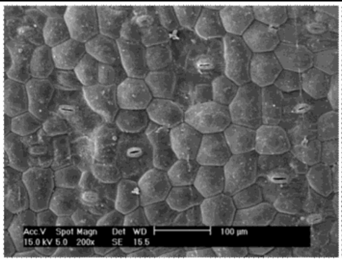	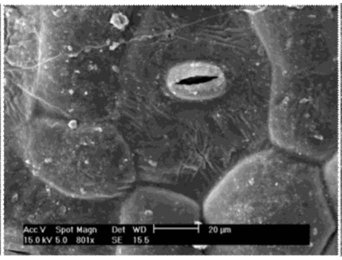	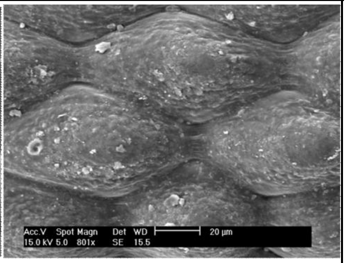

Abaxial leaf epidermis	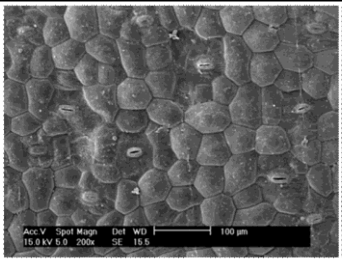	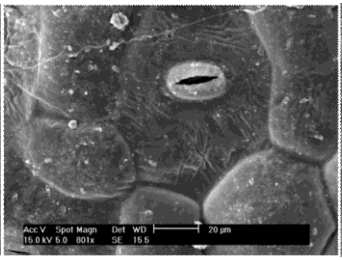	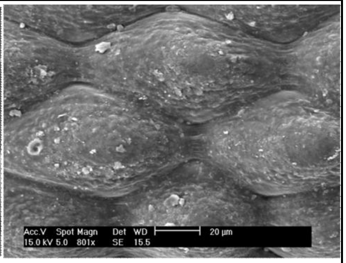
Adaxial leaf epidermis	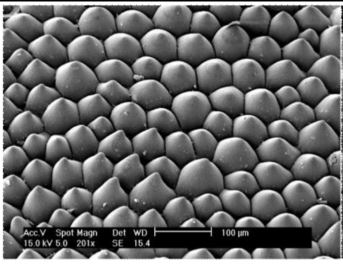	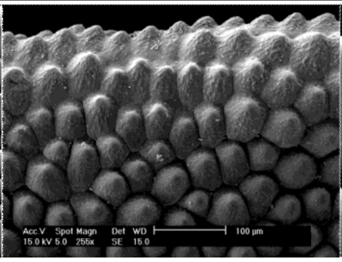	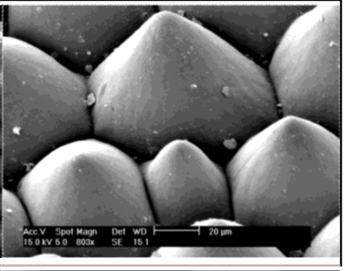
Inflorescence rachis	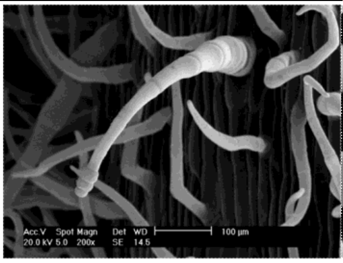	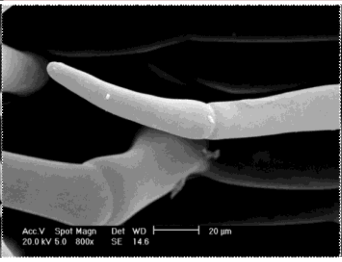	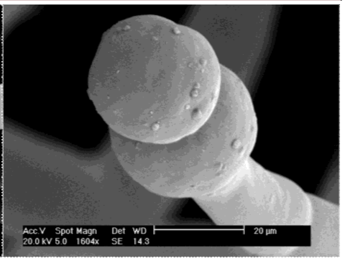
