# Supplementary material for: Floral and Vegetative Micromorphology of Selected Species of Anoectochilus (Orchidaceae): Insights into Trichome Diversity and Peloria
Source: Biology (Basel). 2026 Aug 8;15(16):1341. doi: 10.3390/biology15161341 (PMC13509326; doi:10.3390/biology15161341)

Table S2. Micromorphology of *Anoectochilus formosanus* Hayata

Floral structures

Structure	SEM Image 1	SEM Image 2	SEM Image 3
Lateral sepal	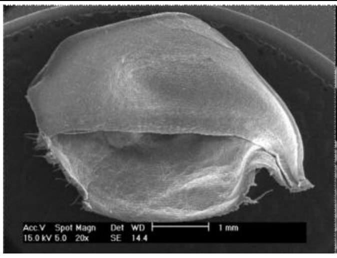	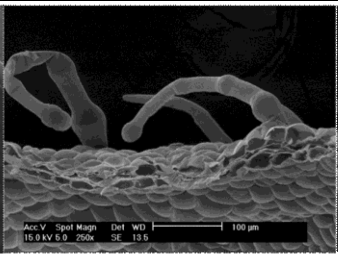	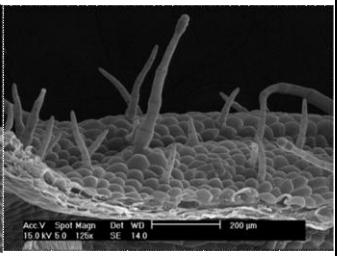
Gynostemium (column)	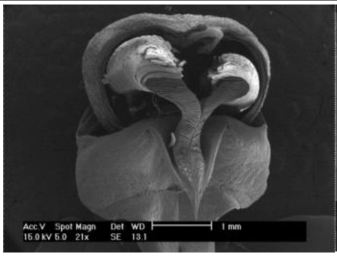	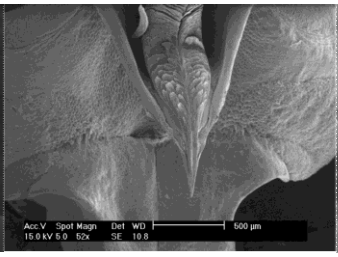	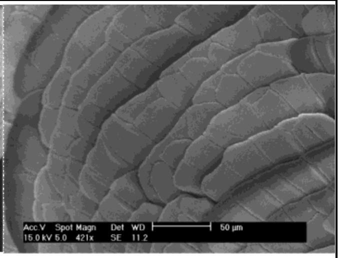
Dorsal sepal	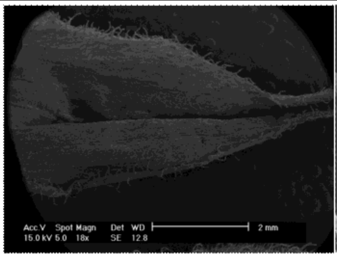	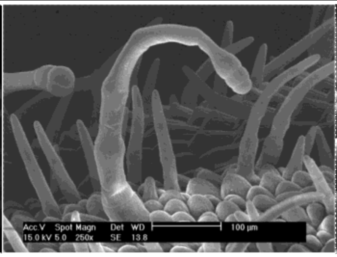	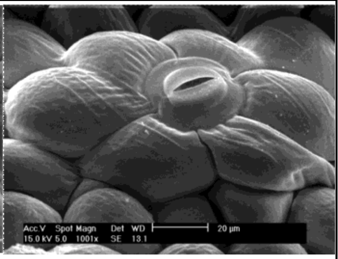
Labellum	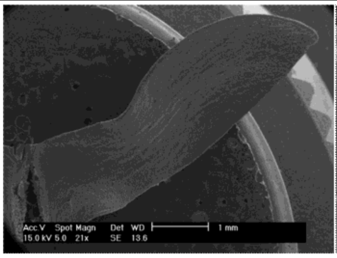	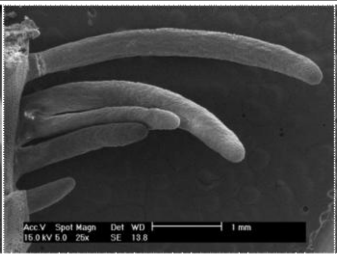	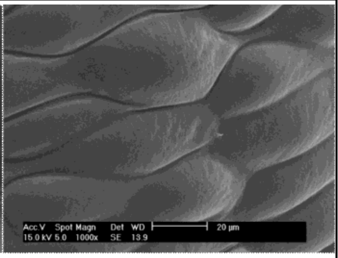
Ovary	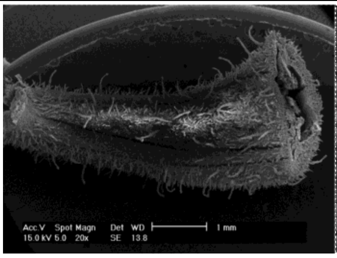	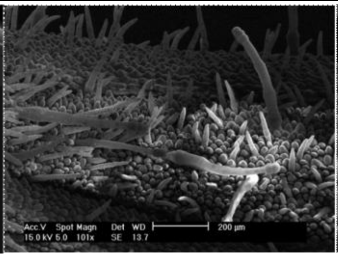	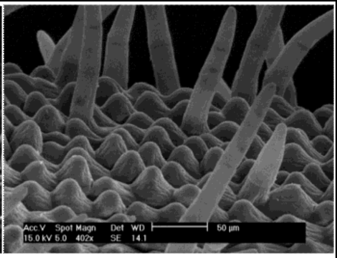

Vegetative structures

Structure	SEM Image 1	SEM Image 2	SEM Image 3
Abaxial leaf epidermis	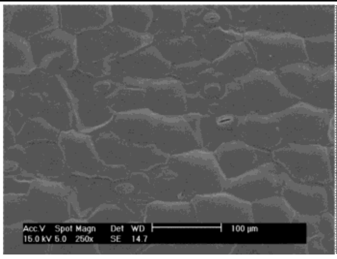	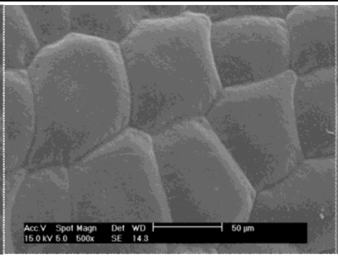	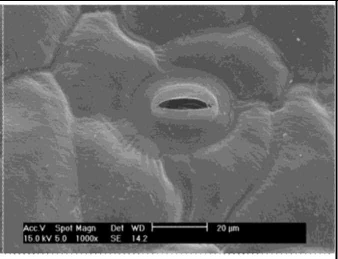

Adaxial leaf epidermis

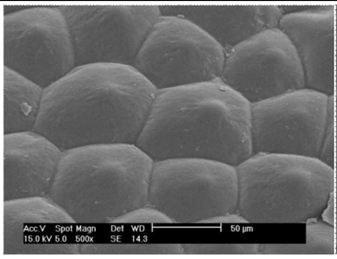

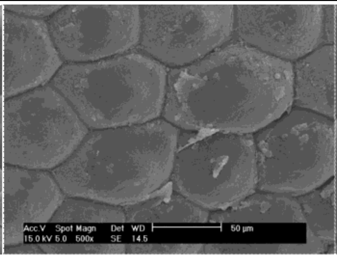

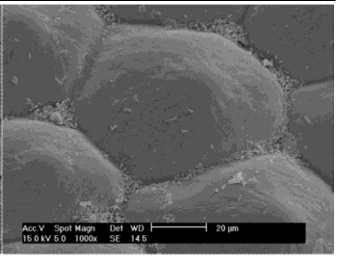

Supplement: Supplementary file 1 [file biology-15-01341-s001.zip › Table S2 A.formosanus.pdf]
